# Water, solute, and ion transport in *de novo* designed membrane protein channels

**DOI:** 10.1021/acsnano.4c11317

**Published:** 2024-12-23

**Authors:** Yuhao Li, Bradley S. Harris, Zhongwu Li, Chenyang Shi, Jobaer Abdullah, Sagardip Majumder, Samuel Berhanu, Anastassia A. Vorobieva, Sydney K. Myers, Jeevapani Hettige, Marcel D. Baer, James J. De Yoreo, David Baker, Aleksandr Noy

**Affiliations:** † Materials Science Division, Physical and Life Sciences Directorate, Lawrence Livermore National Laboratory, Livermore, CA 94550, USA; ‡ Physical Science Division, Pacific Northwest National Laboratory, Richland, WA 99354, USA; ¶ School of Natural Sciences, University of California Merced, Merced, CA 95343, USA; § Institute for Protein Design, University of Washington, Seattle, WA 98195, USA; ∥ Department of Biochemistry, University of Washington, Seattle, WA 98195, USA; ⊥ Structural Biology Brussel, Vrije Universiteit Brussel, Brussels, Belgium; # VUB-VIB Center for Structural Biology, Brussels, Belgium; @ Howard Hughes Medical Institute, University of Washington, Seattle, WA 98195, USA

## Abstract

Naturally occurring membrane protein pores composed of polypeptide chains that fold into 3-dimensional structures dictated by their sequences, can perform a wide variety of functions. Synthetic *de novo* designed membrane pores could open up almost limitless array of possibilities for new functionality. Here we explore water, solute, and ion transport in three *de novo* designed *β*-barrel membrane channels in the 5-10Å pore size range. We show that these proteins form passive membrane pores with high water transport efficiencies and size rejection characteristics consistent with the pore size encoded into the protein structure. Ion conductance measurements and ion selectivity measurements also show trends consistent with the pore size, with the two larger pores showing weak cation selectivity. MD simulations of water and ion transport and solute size exclusion are consistent with the experimental trends and provide further insights into structure-function correlations in these membrane pores.

## Graphical Abstract

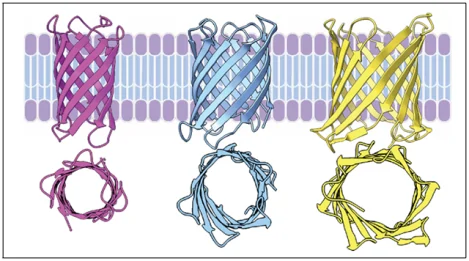

## Introduction

Biological transmembrane channels are some of the most versatile components of the machinery of life. These cellular membrane protein machines can transport or pump a variety of ions, small molecules, and water molecules along or against concentration gradients,^1^ and activate or block transport in response to a voltage stimulus,^2^ ligand binding or mechanical strain,^3^ and do it all with nearly single-species selectivity and efficiency approaching thermodynamical limits.^4^ Remarkably, living systems achieve all this functional diversity using folded polypeptide chains composed of linear sequences of amino acids. Researchers have been trying to replicate this functionality by creating artificial membrane channels using synthetic molecular architectures,^5^ nanotube porins,^6^ and even DNA origami pores.^7^ While some of these examples showed remarkable transport properties,^8^ these approaches have yet to come close to the performance, single-molecule reproducibility, and versatility of biological membrane pores.

An alternative approach to creating efficient transmembrane transporters is to harness the advantages of the folded polypeptide architecture and the power of modern computational design strategies to create *de novo* designed protein units.^9,10^ Recent reports have shown that it is possible to design polypeptide sequences that will fold into *β*-barrel structures that can incorporate into the lipid membranes and form defined transmembrane pores.^11^ This strategy opens up a possibility to design *de novo* membrane channels with a virtually unlimited range of channel sizes geometries and channel functionalities that could exceed the diversity of biological membrane channels.^12^

For this work we targeted a class of passive membrane pores which enable translocation of molecules based on size or molecular weight cutoffs. We built on a previously-reported strategy of using *β*-barrel architectures as a starting scaffold to design pores with inner diameters in a range not extensively explored by nature: 0.5-1.5 nm.^12,13^ In principle, this strategy should also allow sampling of different pore properties such as charge distribution and specific binding sites for different types of ions and molecules, however for this study we have largely restricted our efforts to designing passive pores with controlled inner diameter.

In this work, we explore the transport properties of three examples of such *de novo* protein pores designs that exploit *β*-barrel structure motif to control and vary the pore size. We report a series of transport experiments that establish water permeability of these pores and their size exclusion properties, as well as their ion transport behavior and permselectivity. We show that rational design indeed allows us to manipulate the pore sizes, and that the measured transport characteristics follow the expected trends based on the channel geometry and degree of spatial confinement.

## Results and Discussion

### *De novo* protein design

We built transmembrane *β* barrel (TMB) backbones with water-accessible pores from the ground up using the principles established earlier and demonstrated with the design of 8-stranded TMBs.^11,12^ To adjust pore size, we varied the number of *β*-strands (10, 12, and 14) while maintaining transmembrane span and *β*-strand connectivity. This led to an increase in average *β*–barrel diameter from 16.4 Å (8 strands) to 19.4 Å (10 strands), and 22.8 Å (12 strands) (Figure 1a-c).^12^ Larger *β*-barrel diameters preclude long-range sidechain contacts across the pores, necessitating local encoding of structural properties such as *β*-strand pairing and barrel shape. ^12^ To strengthen the structure and simplify folding, we introduced 2- and 3-residue *β*–hairpins to connect *β*-strands, as these loops support efficient folding of TMBs. Initial designs, created using Rosetta BlueprintBDR, featured cylindrical shapes strained by repulsion between side-chain packing in the barrel lumen.^12^ We also introduced glycine kinks into the blueprint to alleviate strain and facilitate bending of *β*-strands to form corners in the *β*-barrel cross-section. A major challenge for the TMB design is to optimize the folding of the *β*-barrel state within the membrane, while ensuring that folding in water is sufficiently delayed to minimize misfolding and aggregation. Only a combination of these approaches enables successful integration into a membrane bilayer.^12^ Our aim was to design larger TMB nanopores by incorporating local secondary structure frustration. Within the water-accessible pore, we engineered networks of polar residues surrounding the canonical TMB folding motif *Tyr -Gly-Asp/Glu*, enhancing strong local *β*-register defining interactions interspersed with patches of hydrophobic and disorder-promoting residues (*Gly, Ala, Ser* ).^12^ To address the increased hydrophilicity of larger TMBs, we further reduced *β*–sheet propensity by substituting a select number of *β*–branched residues with *Ser* and *Thr* amino acids on the lipid-exposed surface.^12^

To design the overall sequence of pore lining residues, we utilized the standard betanov16 Rosetta score function while adjusting reference energies. This approach resulted in predominantly uniform arrangements of positively and negatively charged amino acids interspersed with intermittent hydrophobic residues. For these initial designs, we did not yet aim to incorporate specific pore sequences that could drastically changes pore transport properties, primarily due to the absence of robust models correlating transport properties with pore charges and atomic distributions.

The design strategy robustness was validated by making several different mebrane-incorporating TMB protein variants with different number of strands and less than 30% sequence homologies.^12^ For the current work, we selected from this library one 8-stranded TMB (TMB2.17, referred to as TMB8 later in the text), one 10-stranded TMB (TMB10_165, referred as TMB10 later in the text), and one 12-stranded TMB (TMB12_3, referred as TMB12) (Figure 1a-c). The pore shapes and sizes encoded into these designs suggested that we expected that TMB8 should be largely impermeable, ^11^ and that TMB10 and TMB12 should show significant water and ion conductance with overall permeability increasing for the larger barrel sizes.

### AFM imaging of *de novo* proteins in supported lipid bilayers

To examine insertion of the *de novo* protein pores into lipid bilayers we used atomic force microscopy (AFM). AFM, which uses a sharp probe tip to scan the sample surface with the nanometer-scale resolution, remains the technique of choice for label-less imaging of soft matter interfaces.^14^ We formed supported (DPhPC:DOPE(70%:30%) lipid bilayers (SLBs) on the mica surface^15^ up to the point when the surface was almost completely covered (see Figure S1). Cross-sectional analysis of the AFM images of SLBs indicated a thickness of approximately 3.8 nm, consistent with prior AFM studies of SLBs on mica.^16^ After we formed the SLB, we introduced the TMB12 protein solution into the microscope fluid cell and took the AFM images (Figure 1d). These images revealed that after we introduced the protein, the previously featureless surface of the SLB began to show small dots, corresponding to the *de novo* pores inserted in the bilayer. The number of these dots also increased when we exposed the SLB to progressively higher concentrations of the protein (Figure 1d, i-iv). Time-lapse images and kinetics of TMB12 insertion into the SLB (Figure S2) also were consistent with the protein pores insertion into the SLB. AFM images of the TMB8 and TMB10 protein pore insertion were similar to those of TMB12 as well (Figure S3). TMB12 AFM images indicated that the end of the protein inserted into the membrane protruded on average 0.5 nm above the bilayer (Figure S4). Considering the average measured thickness of the bilayer (~ 3.5 nm) and the estimated thickness of the solvation layer between the bilayer and mica surface (~ 1 nm), we can infer that the ~ 4 nm tall *de novo* membrane pores were not in direct contact with the mica surface, eliminating the possibility of protein denaturing due to mica surface interactions; thus, we concluded that the AFM images capture the true topography of the SLB membrane with *de novo* designed protein pores.

### Water transport measurements

To assess the water transport efficiency of *de novo* protein pores we used an osmotically-induced transport assay. We have exposed lipid vesicles containing protein pores in the lipid shell, prepared by surfactant-assisted reconstitution procedure (see Methods for details), to a solution of an osmolyte in a stopped-flow spectrometer (Figure 2a). The osmotic gradient induced water efflux from the vesicles, shrinking their size, as assessed from the increase in the light scattering signal from the vesicles (Figure 2b-d). The comparison between the light-scattering kinetics recorded for vesicles containing the *de novo* protein pores and control experiments with bare vesicles (Figure 2b-d) showed that in all cases the presence of the protein pores was associated with faster water efflux kinetics, indicating high water permeability of those pores. Notably, the larger the protein pores were the bigger difference the stopped-flow traces showed relative to the control trace, indicating that larger pore diameters corresponded to higher water permeability. We also extracted the single protein water permeability values from the stopped-flow kinetic curves (see Methods for details). These calculations assumed that all added membrane protein had been incorporated into the vesicles; therefore, our protein water permeability values represented a lower bound estimate. All proteins that we tested showed water permeability values on the order of 10^−15^-10^−13^ cm^3^/s which were comparable to the literature values of some of the common biological membrane channels (Figure 2e, see also SI Table S1). As expected, unitary water permeability values increased with the number of strands in the *β*-barrel and the corresponding increase in the protein pore size.

The smallest *de novo* pore, TMB8, was expected to not transport water instead however, it showed unitary channel permeability that was two orders of magnitude smaller than that of aquaporin 1 (AQP1) and KcsA channels (both of which feature single-file water arrangement). Interestingly, TMB8 water permeability was comparable to that of another single-file channel, gramicidin A (Figure 2e), which has a transient single-file water pore.^17^ Molecular simulations reveal structural features that could be responsible for this similarity, which we will discuss in the next section. TMB8 also showed almost identical water efflux kinetics when we replaced larger sucrose osmolyte with the NaCl osmolyte (Figure S5a), indicating that TMB8 has high salt rejection, again conforming to our predictions based on the TMB8 structure.

### Water transport simulations

To gain more insights into water transport through the *de novo* protein pore, we performed unbiased atomistic molecular dynamics simulations (see Figure S6 for internal structures and sizes). Surprisingly, we found that TMB8 protein exhibited pronounced gating (see Figure S7 for representative structures of the open and closed states of the gate). This gate is composed of four residues, Phe, Tyr, Met, and Gln, which allow for intermittent single-file water channel arrangement of 4-5 molecular lengths. ^18^ As this gating behavior is likely to dominate the solvent transport behavior, we focused our simulations on the TMB10 pore, the smallest protein with a continuous pore. The calculated free energy profile for water molecules based on the density profile (Figure 2f) highlights the absence of a significant barrier along the protein pore for the 10-strand *β*-barrel design. Unsurprisingly, the diffusivity along the z direction (Figure 2g) also did not show significant deviations from the calculated bulk water diffusivity of 6.1 × 10^5^ cm^2^/s (we note that this number is in good agreement with literature values for the TIP3P water model used here^19^).

We then calculated the water permeability $p_{f}$ value for the TMB10 channel using non-equilibrium MD simulations where we applied hydrodynamic pressure, which can be directly related to the experimentally applied osmotic pressure (see Methods and Figure S8 - S10). Indeed, the $P_{f}$ value of 0.9 ± 0.2 × 10^−13^ cm^3^/s shows excellent agreement with the 1.3 ± 0.5 × 10^−13^ cm^3^/s value measured in the experiment (Figure 2e, see also SI Table S1 for further discussion on solvent transport simulation results).

### Size exclusion measurements

Osmotically-triggered water efflux experiments also provide an opportunity to investigate size exclusion properties of the protein pores. These experiments are based on the observation that when the pore does not reject the osmolyte perfectly it results in a more complicated kinetics of the vesicle size change.^20^ In this situation the vesicles go through an initial fast water efflux stage when the water is rapidly expelled from the vesicle lumen and the vesicle shrinks, producing a rapid increase of the scattering signal. However, a concurrent slow diffusion of the osmolyte through the pore into the vesicle lumen causes a vesicle to eventually backfill, partially restoring its size and producing a characteristic slow decrease in the light scattering signal at longer timescales (Figure 3a).

We have exploited this effect to quantify the rejection of a series of neutral osmolytes by the protein pores and determine their size exclusion characteristics. For these experiments we used a ladder of sugars and PEGs of different sizes ranging from 150 Da to 1000 Da, and then exposed vesicles with *de novo* protein pores TMB10 and TMB12 (with the TMB8 pore being too small to transport glycine, the smallest molecule among all osmolytes, see Figure S5b) to a 100 mM solution of each osmolyte and captured light scattering kinetics over the longer time scales (see Methods for details). These kinetics showed a variety of behaviors. When the osmotic water transport through the relatively small TBM10 pore triggered by raffinose, a large 504 Da trisaccharide osmolyte, the kinetics showed rapid water efflux with subsequent saturation of the scattering signal (Figure 3b), indicating that TMB10 effectively rejected raffinose. In contrast, when we changed osmolyte to a smaller disaccharide sucrose, the kinetics showed signs of the water backfill process, which became very apparent when we used even smaller monosaccharide glucose and N-acetyl-D-glucosamine (NAD) osmolytes (Figure 3b).

Water efflux kinetics recorded with a larger TMB12 pore showed an even more extreme dependence on the osmolyte size, where all osmolytes smaller than 1000 Da showed clear signs of this water backfill process (Figure 3c). The kinetics recorded with smaller osmolytes, such as glycine, arabinose, glucose, and sucrose not only showed pronounced backfill kinetics, but also did not even display a clear signature of the initial water efflux (Figure 3c), indicating that these osmolytes were diffusing into the vesicle lumen almost immediately after vesicle and osmolyte solutions were mixed.

These kinetic data allowed us to construct the size exclusion curves for each protein and determine its molecular weight cut-off (Figure 3d). As expected, the 12-stranded *β*-barrel TMB12 showed a larger size cutoff at 466 Da than the smaller 10-stranded *β*-barrel TMB10 at 281 Da. Notably, these size cut-off values are consistent with the pore sizes that we predicted based on the protein structure (Figure 3e).

### Modeling of solute transport.

To gain more insights into the process of solute transport through the *de novo* protein pore, we have performed molecular dynamics simulations of the proteins and differently sized solutes. To screen for size exclusivity, we calculated potential of mean force (PMF) profiles through the pore using the methodology described by Bodrenko *et al.* in 2019^21^ (see Methods for details). This approach reduces the PMF down to steric contributions based on solute size and the fluctuations of the pore. This approximation allows for the rapid screening of pores for size exclusivity without having to simulate in-depth interactions between all combinations of solute and pore. Calculated fluctuations in pore radius in the narrowest part of the protein pores (Figure 3e), show two constriction regions, with TMB12 pore being around 2Å wider than TMB10 at its narrowest regions. This additional space allows larger solutes to permeate, which is apparent when comparing the probability distribution functions for the pores and solutes (Figure 3f). The resulting PMF bands (Figure 3c,g) show TMB10 rejecting solutes larger than glucose, which agrees with the experimental results that indicate greater than 90% rejection for solutes the size of NAD and above.

For TMB12 (Figure 3h) we would expect the PMF for sucrose to be slightly below that of arabinose in TMB10 based on experimental results. The highest peaks come close to matching this expectation at ca. 4.2 kcal/mol and ca. 7 kcal/mol, respectively. An additional PMF calculated from constrained MD simulation for glucose through TMB10 (Figure 3g) validates the steric approximation used for the other PMF calculations, as the height and shape of the free energy barriers are similar for both methods with a maximum barrier around ca. 12 kcal/mol; see also SI for further discussion (Figure S11). This comparison suggests that steric hindrance effects dominate the size exclusion properties and that our modeling approach provides an effective way to screen solutes for size exclusion in protein nanopores rapidly.

### Ion conductance measurements.

We also investigated ion transport in *de novo* designed protein channels using a droplet interface bilayer (DIB) system (Figure 4a, inset). This approach, often used in membrane channel studies,^22^ involves forming two lipid monolayer-encased liquid droplets (1 M KCl, pH 7.5) inside an oil bath and bringing them in contact, which forms a small lipid bilayer region in the droplet contact zone. When we introduced TMB10 and TMB12 protein channels into these droplets while monitoring ion current across the droplets, we observed rapid ion current jumps corresponding to spontaneous incorporation of these pores into the bilayer membrane (Figure 4b,d). In contrast, TMB8 proteins never showed conductance jumps, indicating that its pore was too small to enable ion passage, consistent with its behavior observed in the osmotic shock experiments. The histogram of individual conductance jump values of TMB12 displayed a wide distribution (Figure 4a), with a peak conductance value of 0.31 ± 0.02 nS. TMB10 exhibited a narrower distribution in its conductance histogram (Figure 4c), with the most prominent peak position indicating a unitary channel conductance value of 0.19 ± 0.01 nS. Measured unitary pore conduc-tance values (Figure 4b, inset) follow the expected trend in the protein pore diameters and compare well with the previously reported conductance values from the the planar lipid bilayer measurements.^12^ The unitary conductance values measured in this study and adjusted to the same 0.5M electrolyte concentration used by Majumder et.al.^12^ were 0.10 nS vs. 0.12 nS for TMB10, and 0.15 vs 0.25 nS for TMB12. As our data are based on much larger number of individual conductance jumps for each protein, we believe that it represent a more accurate quantification of the ion conductance values for these pro-teins. Some of the discrepancy between the two reported sets of values are likely related to a commonly observed non-linear ion conductance scaling vs ion concentration for nar-row membrane channels.^23-25^ The wider conductance value distribution observed for the larger TMB12 pore may also indicate that TMB12 folds into a less rigid structure than the smaller TMB10 channel.

### Ion selectivity measurements.

To assess the ion selectivity of these protein channels, we used reversal potential measurements. In this protocol we measure a current-voltage (I-V) curve for a channel inserted into a bilayer between two droplets that contained different concentrations of KCl electrolyte. In all control experiments when both droplets were filled with identical electrolyte concentrations, the I-V curves of both TMB10 and TMB12 proteins passed through the origin (Figure 4e). The conductance values derived from these I-V curves also aligned closely with the peaks of the conductance jump histograms. Both TMB12 and TMB10 exhibited a rectification effect for ion transport with a factor of ~2. Introducing ion concentration gradient across the two droplets led to noticeable shifts in the I-V curves (Figure 4f). The measured values of pure osmotic voltage offsets (obtained after correcting for the redox potential of the electrodes), allowed us to determine the channel’s conductivity between K^+^ and Cl^−^ ions. Both protein channels displayed only weak selectivity between these ions at neutral pH, with a K^+^/Cl^−^ selectivity of only ~1.5, with both proteins showing statistically indistinguishable selectivity values. Error bars were again larger for the TMB12 pore, likely due to the larger pore size or its greater flexibility.

### Ion transport modeling.

To investigate the mechanism of ion transport and the observed weak cation preference in these channels, we employed constrained molecular dynamics (MD) simulations to compute the free energy profiles for the translocation of individual *K*^+^ and *Cl^−^* ions within the channels. We chose the starting structures to contain only one ion in the channel to estimate a single ion free energy. The free energy profiles correlate with the differences in the electrostatic potential along the channel surface (Figure 4g). Although the TMB10 channel maintains an overall neutral charge at neutral pH, with most charged residues located internally, we can identify two zones that align with the narrowest sections of the channel and which appear to be critical for ion transport. The first constriction zone is predominantly neutral, while the second displays a minor positive charge surplus. Unlike TMB10, the overall charge of TMB12 under neutral conditions is negative (−3) due to a larger number of acidic groups, which leads to a negatively charged region within the channel. This effect can be seen in the electrostatic potential of the channel surface (Figure 4h, inset image).

The calculated free energy profiles for TMB10 (Figure 4g), reveal that both the positive and negative ions encounter maximum energy barriers of ca. 4 kcal/mol along the channel. Notably, the profile for both *K*^+^ and *Cl^−^* ions suggest that they encounter those local energy barriers, two main constriction sites inside the channel (Figure 4g). Interestingly, both ions exhibit repulsion at the first barrier region, indicating that confinement may be the primary factor influencing the ion passage at that site. In contrast, the second site at 5.15 nm along the channel presents a potential energy barrier for the *K*^+^ ion and a local energy minimum for the *Cl^−^* ion, suggesting that the nature of the barrier at this site is primarily electrostatic. We also note that the maximum value of the energy barrier along the channel for *Cl^−^* ions is 0.45 kcal/mol higher (4.25 vs. 3.80 kcal/mol) than for *K*^+^ ions. This energy difference predicts that the *K*^+^ ions should transport ca. two times faster than *Cl^−^* ions, which correlates reasonably well with the measured *K*^+^/*Cl^−^* selectivity value of 1.5. For further discussion see SI and figure S12.

For the TMB12, the effect of the negatively charged region (Figure 4h, inset image) is directly correlated with a local minimum in the free energy for *K*^+^, and a corresponding barrier for the *Cl^−^* ion (Figure 4h). The qualitative trend for ion selectivity can be seen in the barriers, but the absolute values largely overestimate the experimentally observed ratio. The effect of ion-ion interactions within the channel cannot be ruled out, as even in the free 1-microsecond simulations, multiple ions are found within the TBM12 channel. This is not the case for the TMB10, due to the smaller radius and larger observed barriers. The TMB8 is not expected to transport ions and the PMF is only calculated to estimate the barrier to cross the gate region, see SI Figure S13. The high barrier of about 15 kcal/mol for both ions at the gating region suggests that ion transport is an unlikely event under equilibrium conditions.

## Conclusions

We have combined experimental measurements and MD simulations to study water, solute and ion transport properties of three *de novo* designed TMB membrane pores composed of 8, 10 and 12 *β*-strands. Vesicle-based stopped-flow experiments show that both proteins form passive membrane pores with high water permeability that scales with the pore size. Solute exclusion experiments utilizing a size ladder of different sugar and PEG molecules indicate size exclusion characteristics consistent with the designed pore size. Ion transport properties of the TMB proteins were also consistent with the pore sizes, showing weak cation selectivity and ion conductance values that were consistent with those of biological channels in the similar pore size range. MD simulation results are consistent with the observed trends and provide molecular-level detail of how the designed structures of the pores shape their transport properties.

Our results point to an almost limitless array of possibilities that this de novo design strategy brings to membrane pore engineering, relative to the other artificial membrane channel platforms (Table S3). Our detailed analysis of pore sequence/function relationship informs rational design and could eventually enable the design of pores with tailored functionality. Different peptide sequences can encode different pore shapes and define the precise placement of chemical functionalities inside the channel, opening up opportunities to engineer precise pore selectivity that can match and perhaps exceed that of biological pores. Incorporation of outer membrane domains with vestibule functionality and adding external or internal binding sites could further extend functionality and enable gated transport. By suitably designing the outer surface of the pores, it should be possible to promote oligomerization in the membrane, enabling the generation of biomimetic membranes with tightly-packed pore arrays that could deliver breakthrough separation precision and efficiency.

## Methods

### *De novo* protein design, synthesis, and purification

Gene-constructs as designed^13^ were ordered from IDT (Integrated DNA Technologies) in a T7 expression vector (pET29b) with Kanamycin resistance. The proteins were expressed using a standard protocol as previously published.^12,13^ Briefly, an auto-induction medium was used to grow and induce cells over a 24-hour incubation period at 37°C. Cells were harvested and lysed using sonication, and the cell pellets with inclusion bodies were washed extensively with multiple rounds of sonication and centrifugation in different detergents such as Triton X-100 and Brij-35 (purchased from Sigma-Aldrich). Finally, the washed inclusion body pellets were dissolved in 6M GndCl (Guanidium Hydrochloride) solution and refolded in a buffer containing 2 CMC (0.1% w/v) of DPC (Dodecyl Phosphatidylcholine purchased from Anatrace) detergent at a final concentration of 5-10 *μ*M by direct dilution from GndCl buffer. The refolded proteins were then concentrated to less than 1 mL and run on a size-exclusion chromatography column (S200 from Cytiva) and the appropriate peak was used for further downstream experiments. Initial characterisation of the pores included a Circular Dichroism (CD) melt and subsequent test for pore formation in planar lipid bilayer membranes.^13^

### Vesicle preparation and protein reconstitution

The lipid solutions for AFM were prepared according to the following protocol. 1,2-diphytanoyl-sn-glycero-3-phosphocholine (DPhPC) and 1,2-dioleoyl-sn-glycero-3-phosphoethanolamine (DOPE) lipids) were purchased from Avanti Polar Lipids, Inc. These lipids were combined in a 70:30 ratio and subsequently dried under vacuum overnight. The resulting dried powder was rehydrated with a salt buffer to achieve a concentration of 5 mg/mL. The salt buffer composition included 150 mM NaCl, 5 mM CaCl_2_, and 20 mM Tris-HCl at pH 7. The rehydrated solution underwent five freeze-thaw cycles, leading to a uniform milky appearance, indicative of the formation of multilamellar vesicles. In the final step of lipid preparation, the solution was extruded at least 20 times using a Mini-Extruder kit (Avanti Polar Lipids Inc., AL, USA) equipped with a 100 nm filter membrane (Avanti Polar Lipids Inc., AL, USA). This extrusion process resulted in the formation of small, unilamellar vesicles (SUVs). Subsequently, the solution was diluted with 150 mM NaCl to achieve a concentration ranging from 0.02 to 0.1 mg/mL, depending on the specific experiment. Prior to use, all glassware and components underwent thorough cleaning by sonication with ultrapure water, followed by isopropyl alcohol, and water again, each step lasting 20 minutes.

The proteoliposomes for water and solute permeability measurements were prepared through the surfactant-assisted reconstitution. Initially, 32 mg of DOPC lipid in chloroform was introduced into a glass vial, and subsequent to the solvent evaporation using a rotary evaporator, further drying was accomplished in a vacuum desiccator chamber. The resulting dried lipid film underwent hydration with 1 mL of buffer (10 mM HEPES, 100 mM NaCl, pH = 7.5) at room temperature for a duration of 30 minutes. To ensure the formation of large unilamellar vesicles (LUVs), the samples underwent 7 cycles of freeze-thaw treatment. Following this, the LUVs were extruded at least 11 times through 200 nm pore-sized polycarbonate track-etched membranes, utilizing a mini-extruder (Avanti Polar Lipids).

Subsequently, 250 μL of LUVs were transferred to an Eppendorf tube and combined with surfactant (10 mM HEPES, 100 mM NaCl, 2% Decyl-*β*-D-maltoside (DM), pH = 7.5) to achieve a final concentration of 1% DM. The vesicle-surfactant mixture was left to mix on a nutating mixer for approximately 30 minutes. Proteins were then introduced to the tubes along with the first portion of bio-beads (approximately 100 mg, added after 15 mins), and the mixing continued for an additional 45 minutes. Subsequently, the second portion of bio-beads was added, and the tube was left on the nutator for at least another hour. Finally, the proteoliposomes were separated from bio-beads, and the vesicles underwent extrusion for additional 11 times to ensure an evenly distributed size for measurement. For negative control, we added the same volume of buffer to solubilize proteins to the tube, the rest procedures are the same. The diameter of the LUVs was monitored by dynamic light scattering using a Zetasizer Nano ZS90 (Malvern Instruments), with the polydispersity index (PDI) consistently maintaining a value around 0.1.

### Atomic force microscopy imaging

Supported lipid bilayers for AFM imaging were formed by fusing lipid vesicle onto freshly cleaved muscovite mica. Briefly, the respective solutions were pipetted onto a freshly cleaved mica substrate and allowed to equilibrate for 30 minutes before imaging. This process promotes the spontaneous fusion of small unilamellar vesicles (SUVs) to the mica substrate. The morphology of lipids and membrane proteins was examined using a Cypher ES atomic force microscope (Oxford Instrument, Asylum Research, Santa Barbara, CA, USA) at room temperature (25 ° C) employing amplitude modulation AFM (AM-AFM). Raw height AFM images underwent processing using Gwyddion SPM data analysis software. The AFM probe used was SNL-10C (Bruker AFM probes) with a spring constant of 0.24 N/m and a resonance frequency of 70 KHz. To minimize tip-sample interaction and avoid scanning interference, the ratio of the set-point to free amplitude was typically maintained above 0.9.

### Water and solute permeability measurements

Water permeabilities of proteoliposomes or pure LUVs were measured using a stopped-flow fluorescence spectrometer (SFM2000; MOS-200, Bio-Logic) with an excitation wavelength of 546 nm and a 488 nm EdgeBasic long-pass edge filter before the PMT module. LUVs were prepared with an intravesicular solution of 20 mmol/L HEPES, 100 mmol/L NaCl at pH 7.5. During experiments, LUVs solutions were rapidly mixed 1:4 with a hypertonic solution containing 20 mmol/L HEPES, 100 mmol/L NaCl, and 100 mmol/L solute of interest (pH 7.5). The intensity change of scattered light were monitored at 90° at a wavelength of 546 nm. At least three traces were averaged for each data point. We recorded the stopped-flow time trace for 1 second to observe water transport and extended the recording to 4 seconds to observe solute influx. Osmotic water permeability of LUVs, ($P_{f}$ ), in units of cm/s were then calculated using the following expression:^26^

(1) $$P_{f}=\frac{r_{o}}{3\cdot \nu_{w}\cdot \tau }\cdot \frac{c_{in}+c_{out}}{2\cdot c_{out}^{2}}$$

where $r_{o}$ is the LUV radius, τ is the time constant determined from the kinetic curves, $\nu_{w}$ is the partial molar volume of water, and $c_{in}$ and $c_{out}$ are initial solute concentrations inside and outside the vesicle, respectively. The unitary water permeability $P_{w}$ of protein was calculated using the following expression after subtracting the background vesicle permeability:

(2) $$P_{w}=P_{f}^{protein}\cdot \frac{A}{N}$$

where $P_{f}^{protein}$ is permeability of proteoliposome minus background permeability of control vesicles, A is the surface area of the proteoliposomes and N is the average number of proteins per vesicle, estimated from the protein-to-lipid ratio and DOPC lipid numbers of individual vesicle.^27^ The ensuing solute permeability of proteoliposomes, ($P_{s}$), in units of cm/s can be obtained using the following expression: ^28^

(3) $$P_{s}=\frac{r_{o}}{3\cdot \tau_{s}}$$

where $r_{o}$ is the LUV radius, $\tau_{s}$ is the time constant for solute influx determined during the decreased phase of light scattering. For synthetic membrane, the solute flux $J_{s}$ can be written as: ^29^

(4) $$J_{s}=B\Delta C_{s}$$

where B is solute permeability coefficient (cm/s), and $\Delta C_{s}$ is solute concentration difference across the membrane. The rejection of a synthetic polymeric membrane R is defined as:^29^

(5) $$R=(\frac{C_{S,F}-C_{S,P}}{C_{S,F}})\times 100\%$$

where $C_{S,P}$ , $C_{S,F}$ are the solute concentration of permeate and feed side, respectively. Within the context of the solution-diffusion model, R can also be expressed as:

(6) $$R=\frac{\left(A∕B)(\Delta P-\Delta \pi \right)}{1+(A∕B)(\Delta P-\Delta \pi )}\times 100\%$$

where A is the effective membrane permeance to water, ΔP is the pressure difference across the membrane, and Δπ is the osmotic pressure difference across the membrane. We can correlate A with the osmotic water permeability of biological membranes $P_{f}$ : ^30^

(7) $$J_{w}=j_{w}\nu_{w}=\nu_{w}P_{f}\Delta C_{osm}=\nu_{w}P_{f}\frac{\Delta \pi }{RT}$$

(8) $$A=\frac{J_{w}}{\Delta \pi }=\frac{P_{f}\nu_{w}}{RT}$$

where $J_{w}$ and $j_{w}$ are volumetric and mole flux of water, respectively, $\Delta C_{osm}$ is the osmolarity difference, R is the ideal gas constant, and T is temperature. Now that we can obtain A from $P_{f}$ and compare B with $P_{s}$, solute rejection can be calculated as:

(9) $$R=\frac{(A∕B)\Delta \pi }{1+(A∕B)\Delta \pi }\times 100\%=\frac{J_{w}∕P_{s}}{1+J_{w}∕P_{s}}\times 100\%\;\;=\;\;\frac{\nu_{w}P_{f}\Delta C_{osm}}{P_{s}+\nu_{w}P_{f}\Delta C_{osm}}\times 100\%$$

### Droplet interface bilayer measurements of ion conductance

We used a droplet interface bilayer (DIB) setup to measure the ion conductance of protein channel.^22^ Briefly, large unilamellar vesicles (LUVs) with an diameter of ~100 nm, composed of DPhPC, were prepared in a KCl buffer solution (1 M KCl, 10 mM Tris, and 1 mM EDTA, pH 7.5), using the above descibed method. Ag/AgCl electrodes, each 100 *μ*m in diameter and with ball-ended tips, were rendered hydrophilic through a coating of low-melt agarose in KCl buffer (3% w/v). These electrodes were then mounted onto two micromanipulators (NMN-21, Narishige), with one connected to the patch clamp headstage and the other serving as the grounded connection. Within an acrylic chamber containing ~1.0 mL of hexadecane oil, droplets of LUV solution (~300 nL) were placed onto the electrodes using a micropipette. After ~5 min incubation, the droplets were brought together to form bilayers. This process was observed using an inverted microscope (Leica DMi1). Protein channels (either TMB12 or TMB10) were added to the LUV solutions prior to droplet formation. The ionic current was continuously monitored under a potential of 100 mV. TMB8 channel conductance was tested using the protocol described earlier.^12^

For ion selectivity measurements, LUV solutions with differing KCl concentrations were used, specifically 1 M KCl and 0.1 M KCl for the trans and cis droplets, respectively. The Ag/AgCl electrode tips were coated with agarose corresponding to the KCl concentration of each droplet. Post protein channel incorporation, the ionic currents were measured in 10 mV increments to obtain I-V characteristics. The reversal potential ($V_{R}$) was calculated by subtracting the theoretical redox potential ($E_{redox}$) of the electrodes from the zero current voltages using the Nernst equation.

(10) $$E_{redox}=\frac{RT}{F}\text{ln}\frac{\left(a_{cis}\right)}{a_{trans}}$$

where R is the gas constant, T is the temperature, F is the Faraday constant, and a represents the activity of the salt. ^31^ The derived reversal potential was then used to calculate the K^+^ transference numbers ($t_{+}$) for the protein channels with the Henderson equation.^32^

(11) $$V_{R}=(2t_{+}-1)\frac{RT}{F}\text{ln}\frac{\left(a_{cis}\right)}{a_{trans}}$$

The ionic selectivity ratio (SR, cation/anion) was determined using the following equation.


(12) $$SR=\frac{t_{+}}{1-t_{+}}$$

All the measurements were conducted using an Axopatch 200B patch clamp amplifier, which was integrated with a 1550B data acquisition system (Molecular Devices). The data were recorded at a sampling frequency of 20 kHz, with a low band-pass filter of 1 kHz.

### MD simulations

Molecular dynamics (MD) simulations were performed using Gromacs 2022.3^33^ TMB proteins in lipid membranes and solute in water were simulated independently. Initial structures for both TMB proteins in lipid bilayers were built and solvated using the CHARMM GUI^34.35^ Compositions for all simulations are shown in the SI. All systems were neutralized with counter-ions, and ion concentration was set to 0.3 M to mimic biological function. Structures were solvated using TIP3P and equilibrated using standard multi-step CHARMM-GUI minimization protocol before 1 microsecond NPT simulation using Nose-Hoover thermostat^36^ at 303.15K and semi-isotropic Parinello-Rahman^37^ barostat at 1.0 bar. Solutes were also built using the CHARMM-GUI by placing a single solute molecule in the center of the box and then solvating using TIP3P water.^19^ The box dimensions and number of molecules in all simulations can be seen in SI Table S2. Solute systems were equilibrated using steepest descent energy minimization followed by NVT equilibration using velocity rescale thermostat.^38^ Production simulations were performed NPT with the Berendsen thermostat and barostat at 300K and 1 bar for 1 microsecond. ^39^

### Solute transport simulations in de novo designed proteins

TMB pore radius was calculated using the HOLE^40^ program with custom atomic radius parameters to match the atomic radii used for the solute radius determination. Values are provided in SI. The solute radius was calculated by aligning the molecule’s largest moment of inertia with the z-axis and projecting the circumference of all heavy atoms on the xz-plane, using the same radii as provided in the SI. The radius was estimated using the Welzl algorithm.^41^ This procedure was implemented in tcl within VMD,^42^ see SI. The potential of mean force (PMF) estimates were determined from an approximation for the steric contribution of a hard-sphere through the cross-sectional area of the pore. The method was implemented based on the recent work by Bodrenko et al.,^21^ and Jupyter notebooks are available in SI.

### Water diffusivity within TMB10 pore

The z-dependant water diffusivity D(z) within the TMB10 pore was calculated using restraint simulations, with a force constant of 100 kJ/*nm*^2^. The z-position of the oxygen atom for selected water molecules was restrained to its z-position relative to the center of mass of the protein. The diffusivity was then calculated following the procedure as described in detail by Awoonor-Williams and Rowley ^43^ as first introduced by Hummer.^44^ The diffusivity D(z) is related to the variance of z and the correlation time (τ ) by:

(13) $$D(z)=\frac{var(z)}{\tau },$$

where τ is calculated from the integral of the position auto-correlation function.

### Water transport simulations in TMB10 pore

The water permeability $p_{f}$ was estimated from non-equilibrium molecular dynamics simulations by applying a hydrodynamic pressure. Simulations were performed by applying an external force to any water molecule within a 4 nm slice using a development version of Gromacs 2016.5 with modified pull-code from Herrera et al.^45^ The starting configuration was taken from the water permeability simulations. A 3 nm vacuum layer was included by increasing the box to ensure a pressure differential, see SI. Ions were removed from the simulation to ensure no ion gradient was introduced. This is possible as the TMB10 has no total charge. Applied forces with force constants of ± 1347 kJ/mol/nm and ± 674 kJ/mol/nm were chosen to correspond to applied pressures of ± 30 MPa and ± 15 MPa, respectively. Simulations were performed in the NVT ensmble for 36 ns each. The first nanosecond was discarded for analysis purposes. Water molecules that were within the pore for any period of time were identified in VMD using a custom TCL script, and the coordinates for each water oxygen was stored at picosecond intervals. Crossing events were identified while correcting for periodic boundary conditions. Crossing slices were analyzed in 1 Å bins to generate statistics, and numbers were normalized per nanosecond based on simulation time. Resulting data are shown in SI. Analysis for determining $p_{f}$ from these simulations can be found in Zhu *et al.* ^46^ The hydrodynamic permeability $l_{p}$ is calculated from the linear regression of the calculated flux $j_{\nu }$ as molecules per nanosecond, and the applied pressure difference ΔP .


(14) $$j_{\nu }=l_{p}\Delta P$$

The osmotic permeability $p_{f}$ is related to $l_{p}$ as:

(15) $$p_{f}=\frac{RT}{V_{w}}l_{p},$$

where R is the gas constant, T is the temperature, and $V_{w}$ is the molar volume of water(18 cm^3^/mol).

### Ion and glucose transport modeling

PMFs for single glucose, *K*^+^, and *Cl^−^* solutes were calculated using constrained molecular dynamics simulations and thermodynamic integration. For TMB10, 44 independent simulations were performed constraining the z-position of the center of mass for each solute in z from 30.08 to 56.58 in 0.5 Å increments. For TMB12, 56 independent simulations were performed, constraining the z-position for each ion in z from 30.06 to 58.06 in 0.5 Å increments. The average force was calculated for each simulation of 15 ns and integrated using Simpson’s rule. The electrostatic potential was calculated using the APBS^47^ software within VMD. Partial charges for the protein atoms are set to the ones found in the topology for the respective system.

## Supplementary Material

DeNovo_Pores_Transport_SI

Supporting Information is available and contains additional AFM imaging data, a table of MD simulation parameters, and additional water transport simulation data and schematic.

## Figures and Tables

**Figure 1:**
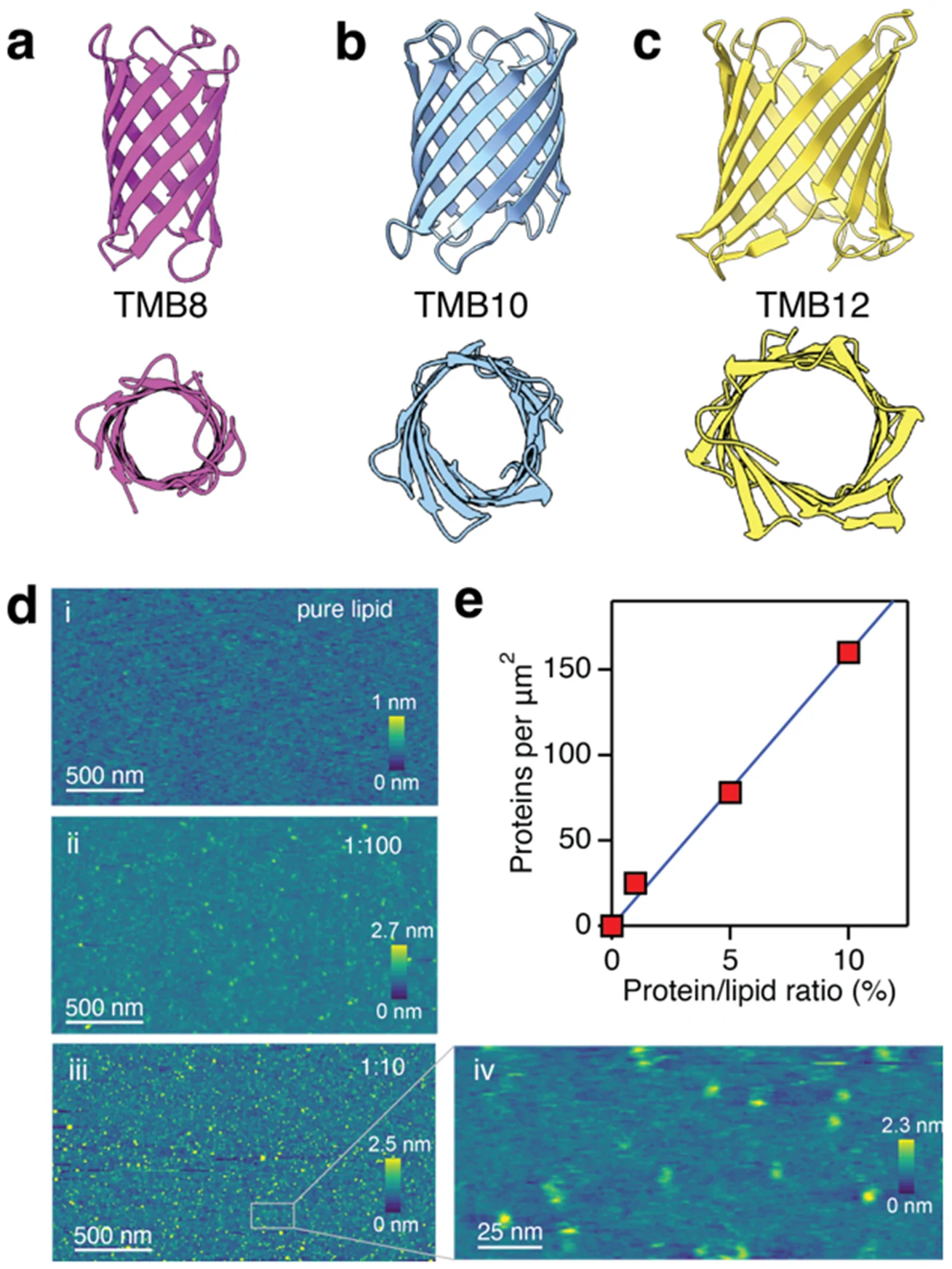
*De novo* designed protein pores. **a-c**. Structures of *de novo* designed (*a*) TMB8 , (*b*)TMB10, and (*c*) TMB12 proteins showing the *β*-barrel structure of the proteins in side view and the shape of the inner pore in top view. **d**. A sequence of the AFM images of a supported lipid bilayer imaged after introducing the TMB12 protein (protein-lipid ratio as indicated on the images). Inset is a zoomed-in part of the image iii, showing individual TMB12 proteins in the lipid bilayer. **e**. Plot of the area density of TMB12 proteins observed in the AFM images as a function of the protein-lipid ratio.

**Figure 2:**
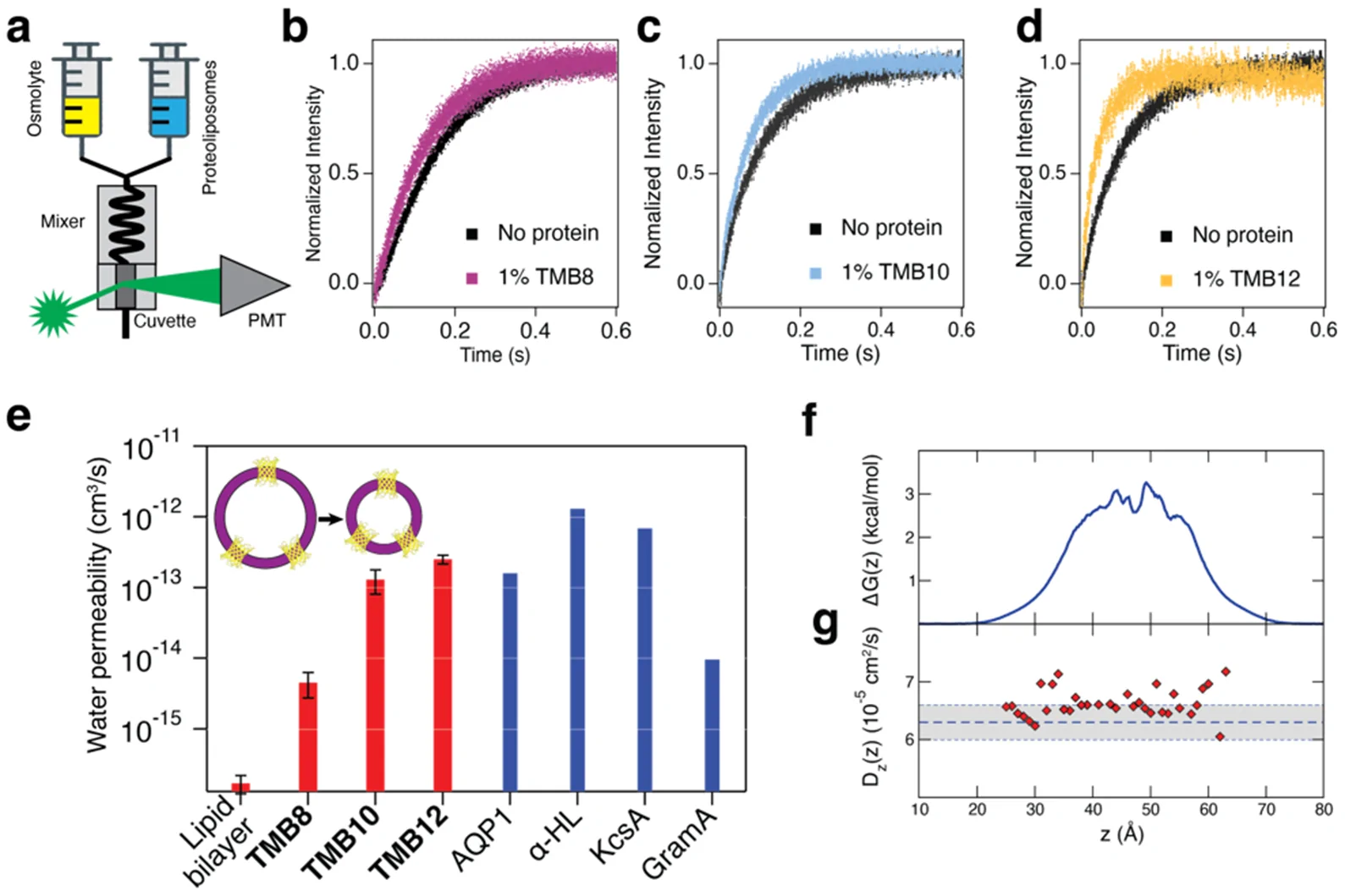
Water permeability of *de novo* designed protein pores. **a**. Schematics of the stopped-flow measurement. **b-d**. Representative normalized stopped-flow traces obtained for vesicles containing 1% of TMB8(*b*), TMB10 (*c*) and TMB12 (*d* ) compared to the control experiments with vesicles without protein pores. **e**. Measured water permeabilities of *de novo* designed proteins (N=3) compared to the literature values of water permeability of biological membrane pores. The baseline water permeability for lipid bilayer was determined from the experimental data by scaling baseline vesicle water permeability to a bilayer area equivalent to the area of the membrane occupied by the TMB12 protein, 3.8 × 10^−14^cm^2^. Inset illustrates the osmotically-induced water transport measurement, where water efflux from the vesicle reduces the vesicle size, increasing the light scattering signal. **f**. Position-dependent free energy ΔG(z) for water molecules computed from the averaged water density profile. **g**. Position-dependent diffusion constant in z-direction $D_{z}(z)$ calculated by restraint molecular dynamics simulation. The blue dashed line highlights the calculated bulk water diffusion constant, and the grey shaded area corresponds to the uncertainty from the bulk estimates.

**Figure 3:**
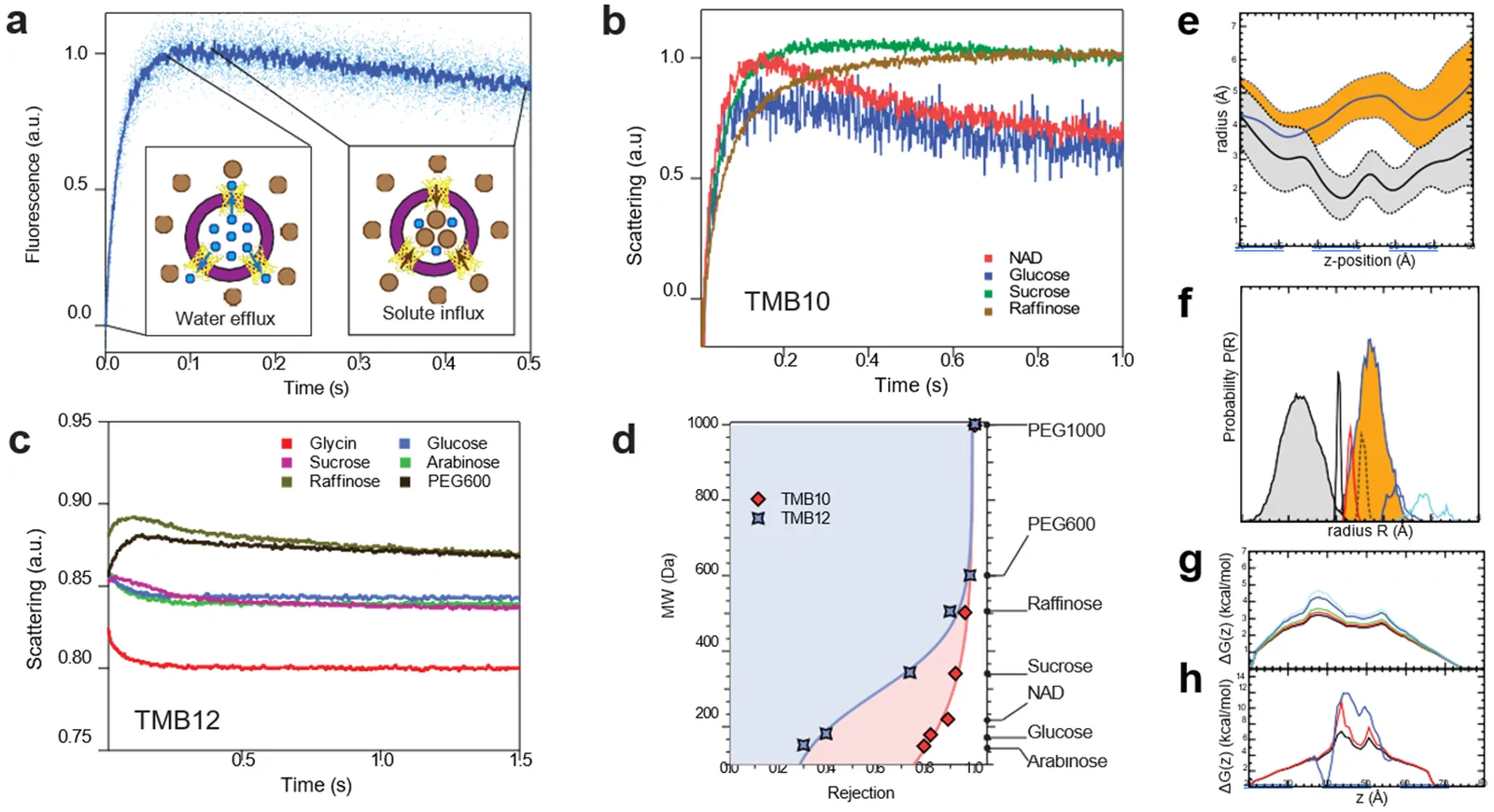
Size exclusion of *de novo* designed protein pores. **a**. Schematics of the two transport regimes observed in stopped-flow measurements when the pore does not fully reject the solute (osmolyte). Vesicles shrink during the initial (fast) water efflux phase and then reinflate due to the slow influx of solute into the vesicle lumen. **b,c**. Representative normalized light scattering signal kinetics observed for vesicles containing TMB10 (*b*) and TMB12(*c*) pores after exposure to different osmolytes. **d**. Size exclusion properties of TMB10 and TMB12 pores. **e**. Ensemble averaged pore radius along the protein channel for TMB10 (black) and TMB12 (blue) from 1*μ*s MD simulation. The grey and orange areas indicate fluctuations. **f** The probability distributions for the radius of the pore in TMB10 (black outline,grey area) and TMB12 (blue outline, orange area) in the area of smallest average radius, and for solutes in solution (arabinose: black, glucose: red, NAD: green, sucrose: blue, raffinose: cyan). **g,h**. Calculated PMFs for different solutes based on hard sphere estimates for TMB12 (*g* ) for (arabinose: black, glucose: red, NAD: dashed brown, sucrose: blue, raffinose: cyan) and TMB10 (*h*) (arabinose: black, glucose: red). For TMB10 a reference PMF for glucose calculated using constraint MD simulations is also shown in blue. All free energies are aligned that the minimum is set to zero.

**Figure 4:**
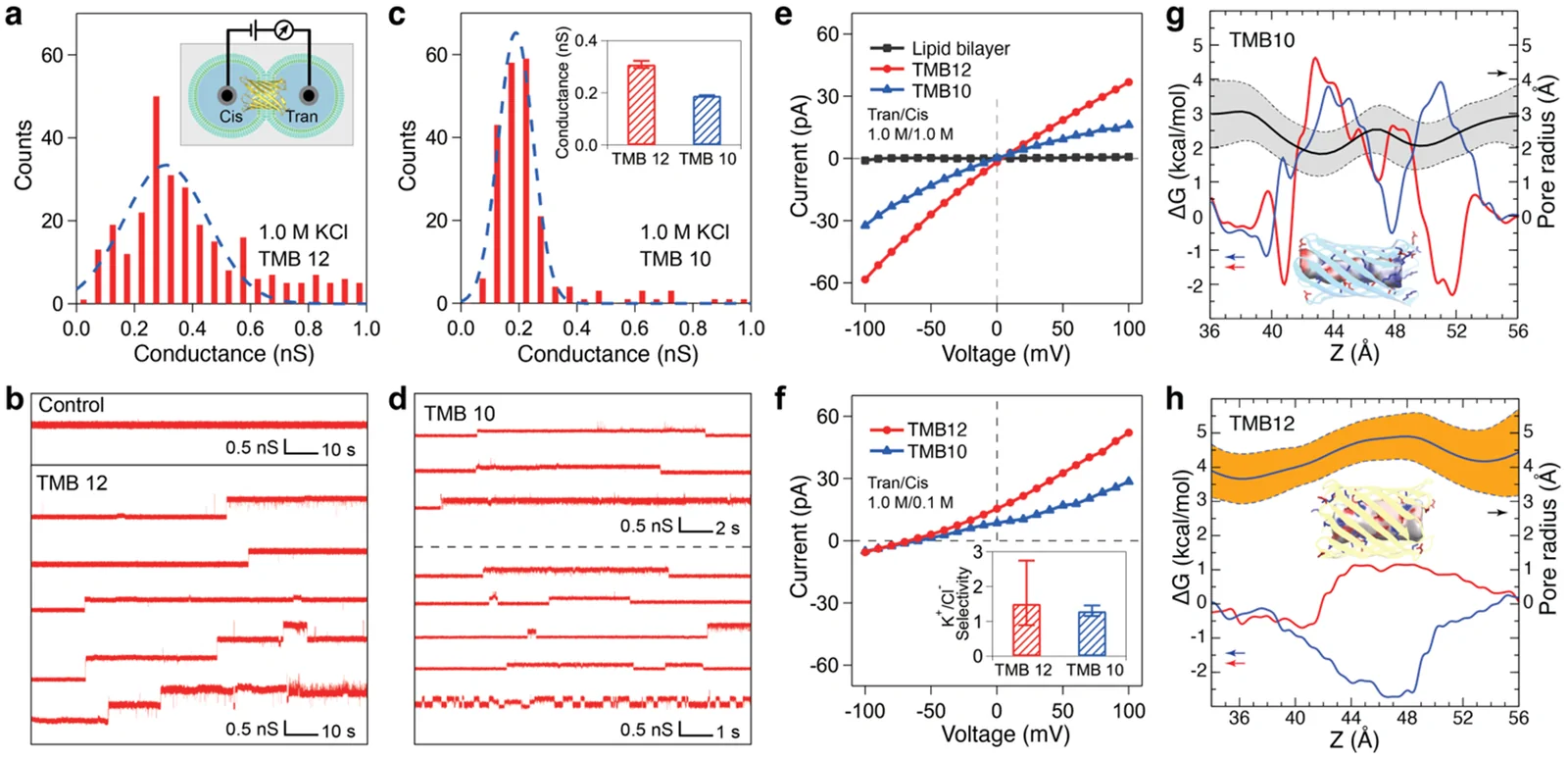
Ion transport in *de novo* designed proteins. **a**. Ion conductance histogram measured for TMB12 (N=300) in a droplet interface bilayer (DIB) setup where the droplets were filled with 1.0 M KCl (1.0 M KCl, 10 mM Tris, 1 mM EDTA, pH 7.5) solution. Blue dash lines indicate best fits to a Gaussian distribution. Inset: Schematic of the DIB setup. **b**. Representative traces showing insertion of TMB12 into the lipid bilayer. **c**. Ion conductance histogram measured for TMB10 (N=213) in a DIB setup where the droplets were filled with 1.0 M KCl (1.0 M KCl, 10 mM Tris, 1 mM EDTA, pH 7.5) solution. Blue dash lines indicate best fits to a Gaussian distribution. Inset: Bar graph of the position of the unitary conductance peak for TMB12 and TMB10. Error bars were determined from the Gaussian fits. **d**. Representative traces showing insertion of TMB10 into the lipid bilayer. **e**. Ionic current-voltage (I-V) characteristics of lipid bilayer (control) and individual TMB12 and TMB10 proteins when the droplets of both trans- and cis- side were filled with 1.0 M KCl (1.0 M KCl, 10 mM Tris, 1 mM EDTA, pH 7.5) solution. **f**. I-V characteristics of single TMB12 and TMB10 when the droplet of trans- side was filled with 1.0 M KCl solution and cis side was filled with 0.1 M KCl solution (0.1 M KCl, 10 mM Tris, 1 mM EDTA, pH 7.5). Inset: K^+^/Cl^−^ ion selectivity of TMB12 (N=13) and TMB10 (N=18) determined from reversal potential measurements. **g,h**. Free energy of Cl^−^ (red) and K^+^ (blue) ion transport through the TMB10 (*g* ) and TMB12 (*h*), estimated using thermodynamic integration. The pore radius is shown as in Figure 3e for comparison. Inset images show electrostatic potential along the pore channel, where basic side chains are shown in blue and acidic in red. The channel surface was calculated using the HOLE program, see Methods for details.
