# Supplementary material for: Water, solute, and ion transport in de novo designed membrane protein channels
Source: ACS Nano. Author manuscript; Available in PMC 2026 Nov 2. (PMC13725935; doi:10.1021/acsnano.4c11317)
Supplement: Supplementary file 1 [file NIHMS2209697-supplement-DeNovo_Pores_Transport_SI.docx]

SUPPLEMENTARY MATERIALS for:

Water, solute, and ion transport in *de novo* designed membrane protein channels

Yuhao Li, Bradley S. Harris, Zhongwu Li, Chenyang Shi, Jobaer Abdullah, Sagardip Majumder, Samuel Berhanu, Anastassia Vorobieva, Sydney K. Myers, Jeevapani Hettige, Marcel D. Baer,*^∗^* James J. De Yoreo,*^∗^*

David Baker,*^∗^* Aleksandr Noy*^∗^*

*^∗^*Correspondence to:

Marcel Baer [(*marcel.baer@pnnl.gov*](mailto:(marcel.baer@pnnl.gov) ), James De Yoreo [(*james.deyoreo@pnnl.gov*](mailto:(james.deyoreo@pnnl.gov) ), David Baker [(*dabaker@uw.edu*),](mailto:(dabaker@uw.edu) and Aleksandr Noy [(*noy1@llnl.gov*](mailto:(noy1@llnl.gov) ).

Figure S1: **Supported lipid bilayers (SLBs) on mica. a-d.** AFM images of SLBs on mica deposited from different liposome concentration solutions:

(*a*) 20 *µ*g/mL; (*b*) 45 *µ*g/mL; (*c*) 62.5 *µ*g/mL; and (*d* ) 87.5 *µ*g/mL. **e-f**. AFM image of SLB (*e*), and the height profile (*f* ) corresponding to the white line in the AFM image in (*e*).

Figure S2: **Kinetics of the *de novo* protein pore incorporation into the SLB. a-d.** Time-lapse AFM images of SLBs on mica after exposure to the TMB12 protein solution for (*a*) 0 min; (*b*) 5 min; (*c*) 35 min; and (*d* ) 60 min. **e**. Plot of the average number of protein pores per 1 *µ*m^2^ of SLB surface area as a function of incubation time.

Figure S3: **TMB8 and TMB10 *de novo* protein pores incorporation into SLBs**. AFM images of the surface of an SLB with TMB8 and TMB10 protein pores incorporated to the bilayer (seen as bright dots on the bilayer surface). Protein-lipid ratio was 1:100 and the protein was exposed to the SLB for 60 min.

Figure S4: **Topography of the supported lipid bilayers with de novo protein pores**. A histogram of the heights of the protein parts protruding above the lipid bilayer. N=30.

Figure S5: **Water permeability of TMB8 protein**. (**a,b**).Normalized stopped-flow traces obtained for vesicles with and without 1% of TMB8 using NaCl (*a*) and glycine (*b*) as osmolytes.The water permeability with both osmolytes is on the order of 10*^−^*^15^ cm^3^/s, which is in the same magnitude as water permeability using sucrose osmolyte. There is no decay of the light scattering intensity signal after reaching plateau, indicating that TMB8 channel is impermeable toward these osmolyte.

# 1 Protein pore size calculations.

The key design parameter for teh protein pores is the average *β*-barrel diam-eter, which increases from 16.4 ˚A (TMB8) to 19.4˚A (TMB10) and 22.8 ˚A (TMB12). This diameter describes the pore formed by the backbone atoms, as the designs have not been optimized with regard to the residues lining the pore. The inner pore diameter, either predicted or determined via X-ray crys-tallography or NMR, can provide more specific insights. TMB8 is assumed not to have a continuous pore. In contrast, TMB10 and TMB12 are expected to have inner diameters of approximately 5 ˚A and 10 ˚A, with the smallest constrictions being about 3 ˚A and 8 ˚A, respectively. Alternatively, pore radii could be estimated based on single-channel conductance using the cylindri-cal pore access resistance model [1], yielding values of 3.5 ˚A for TMB10 and 5 ˚A for TMB12. While these estimates provide valuable insights, they are indirect. A functional pore under operando conditions would experience fluctuations due to interactions with water, ions, and other solutes, affecting the pore’s shape and size. To better relate structure to function, molecular dynamics simulations can model these dynamic behaviors and environmental influences, providing a more accurate picture of pore function.

To gain more insights into the pore dimensions and their fluctuations we have performed unbiased atomistic molecular dynamics simulations for the three TMBs embedded in a DOPC membrane and background KCl elec-trolyte (Figure S6, see Methods for details).

Even though there is a continuous water channel at the end of the TMB8 simulation, as shown in Figure S6a, we have also observed a gating feature with open and closed states (Figure S7). Multiple open and closing events were observed during the simulation. The residues involved in gating are PHE, TYR, MET, GLN. At this gate we also observed an intermittent single file water channel arrangement of 4-5 molecular length.

Figure S6: **Representative snapshot of protein structure and pore size fluctuation**. Representative snapshot after 1 microsecond MD simula-tion, highlighting internal residues. Blue: positive basic, red: negative acidic, green: neutral hydrophobic, pink: neutral polar. Water molecules and their surface are shown. For a. TMB8 b. TMB8 pore fluctuation, c. TMB10 d. TMB10 pore fluctuation e. TMB12 f. TMB12 pore fluctuation

Figure S7: **Illustration of TMB8 water gating**. TMB8 water gating structure from MD simulation. Internal residues are colored based on charge and hydrophobicity. Blue: positive basic, red: negative acidic, green: neutral hydrophobic, pink: neutral polar. A single file water chain is pointed out in the gating region. Left, closed structure, right open.

# 2 Water transport simulations.

The schematic for the flow simulations can be seen in Figure S8 and the resulting fit in Figure S9. The robustness of these measurements for TMB10 suggests that for this class of membrane pore this method is sufficient for a water transport estimate. This approach can be beneficial when rapidly screening new *de novo* protein designs, as water density estimates could be obtained while simulating for other properties such as pore fluctuations. Wa-ter transport PMFs for TMB8 determined through this method (Figure S10) shows one extremely high peak at roughly 4.2 kcal/mol with the other side being much lower at around 3 kcal/mol. This 4.2 kcal/mol peak corresponds to the hydrophobically gated region described in the previous section. The other region of TMB8 corresponds roughly to the same level of constriction experienced in the TMB10 protein that is described in the main manuscript. The TMB12 protein has a much lower barrier of 2.5 kcal/mol that is rel-atively flat, indicating no significant contributions to the barrier from any amino-acid side chain that lines the inner walls of this protein.

Complementary, the the permeability *P_m_* was calculated as:

1	*L/*2

Z

=

*P_m_	−L/*2

*exp*( ^1^ ∆*G*(*z*))

*^RT^	dz*	(1)

*D*(*z*)

where the position-dependent free energy ∆*G*(*z*) and diffusivity *D*(*z*) for wa-ter molecules in the pore were determined through constrained simulations (see Methods for details), [2] with *R* as the gas constant and *T* as the tem-perature. As described in the main text, the resulting free energy density profile reveals the absence of a significant barrier along the protein pore even for 10-stranded beta-barrel designs and bulk water like diffusivity within the pore. Calculating the water permeability value using the area of the simula-tion box perpendicular to the TMB10 protein (7.225*×*10*^−^*^13^cm^2^) estimated permeability value of 1*.*94 *×* 10*^−^*^13^cm^3^/s which is in excellent agreement with the experimentally measured value of 1.3*±*0.5*×*10*^−^*^13^ cm^3^/s and the value of 0.9*±*0.2*×*10*^−^*^13^ that we determined from forced hydrodynamic pressure simulations.

The PMF estimated from the water density profiles from unbiased 1-microsecond molecular dynamics runs as described in the manuscript for all 3 studied TMB proteins are shown in Figure S10. TMB8 shows a pronounced barrier at roughly 4.2 kcal/mol,corresponding with the position of the gating region. The other region of TMB8 corresponds roughly to the same level of

Figure S8: **Illustration of the non-equilibrium flow simulation**. The simulation box is shown in black outline, oxygen atoms of water molecules in red, lipid atoms in grey, and protein in orange. The highlighted areas in blue and green correspond to regions in which water molecules experience an additional force *F* ^+^ for flow in positive x-direction and *F^−^* for flow in negative x-direction.

1.5

1.0

0.5

Flux (#H_2_O/ns)

0.0

-0.5

-1.0

-1.5

-30	-15	0	15	30

∆P (MPa)

Figure S9: **Hydrodynamic permeability estimation based on the av-erage flux (water molecules per nanosecond), calculated at different applied pressures in non-equilibrium simulations**. The full black cir-cles are averaged over all slices considered and error bars are estimated as standard deviation (SD). The zero point is the flux based on a microsecond equilibrium simulation, and the error is estimated as the standard deviation of blocks of 40 ns in length. The blue dashed line represents linear fit to the data.

Table S1: **Water permeability of *de novo* protein pores**. Water per-meability of *de novo* protein pores estimated from simulation and measured in the experiment.

| Protein pore | PMF (cm^3^*/s*) | Flow (cm^3^*/s*) | Experiment (cm^3^*/s*) |
| --- | --- | --- | --- |
| TMB8 | 6*.*43 *×* 10*^−^*^14^ | - | 4*.*5 *±* 1*.*8 *×* 10*^−^*^15^ |
| TMB10 | 1*.*94 *×* 10*^−^*^13^ | 0*.*9 *×* 10*^−^*^13^ | 1*.*3 *±* 0*.*5 *×* 10*^−^*^13^ |
| TMB12 | 3*.*18 *×* 10*^−^*^13^ | - | 2*.*5 *±* 0*.*4 *×* 10*^−^*^13^ |

constriction experienced in the TMB10 protein (red curve) described in the manuscript. The TMB12 protein has a much lower barrier of 2.5 kcal/mol that is relatively flat, indicating no significant contributions to the barrier from any amino-acid side chain that lines the inner walls of this protein.

As all tested methods for the water permeability for TMB10 showed good agreement with each other it is reasonable to use these resulting water PMFs as an estimate for the permeability rates for TMB8 and TMB12. All values are shown in the SI table S1. TMB12 shows good estimated agreement with the experiments and TMB8 is overestimated, which is to be expected as the method assumes TMB8 is in the water diffusion limit, which it is not as it is likely dominated by water gating.

# 3 Solute transport simulations

As described in the main text, the pore fluctuations for all experimentally studied TMB proteins were determined by unbiased 1-microsecond molecu-lar dynamics simulations using a fully solvated lipid bilayer system. These fluctuations were used to estimate the size exclusion behavior of the TMB proteins for a series of neutral solutes with hydrogen bonding ability similar to water molecules. For this class of neutral solutes, we assume that steric hindrance effects will be the most significant contribution to the transfer free energy, dominating the size exclusion properties. To explore the validity of this assumption, we compare our estimates to fully atomistic and con-strained MD simulations for glucose through the TMB10 channel. Figure S11 compares the glucose PMF from the steric approximation model and the constrained MD simulation.

The atomistic model (blue) shows broader constriction regions, and a

5

4

3

∆G (kcal/mol)

2

1

0	2	4	6	8

### z (nm)

Figure S10: **Water transport PMF determined from unconstrained molecular dynamics water density profiles**. Water PMF calculated from water density profiles for TMB proteins: TMB8 (black), TMB10 (red), TMB12 (blue).

14

12

10

∆G(z) (kcal/mol)

8

6

4

2

20	30	40	50	60	70	80

z (Å)

Figure S11: **Comparison of atomistic MD simulations and hard sphere approximation for glucose transport in TMB10**. Reference PMF for glucose calculated using constraint MD simulations (blue) and HS approximation (black). The free energies are aligned so that the minimum is set to zero.

higher ∆G value inside the pore, suggesting that there are additional molec-ular interactions at play or that neighboring regions of the pore influence the shape of the PMF. However, the peak locations and absolute values of both PMF curves show close agreement. This agreement suggests that for the class of neutral molecules studied here, the steric hindrance approximation provides a very effective prefiltering tool for probing specific molecular cut-offs. This approach enables the usage of a living database of osmolytes that can be used to screen new pores in the time it takes to perform a basic MD simulation to determine pore fluctuations. When combined with the findings in the solvent transport section, this approach allows for the rapid approxi-mation of solvent transport, solute size selectivity, and complex effects such as hydrophobic gating from a single simulation.

# 4 Ion transport simulations.

As we discuss in the main text, for the ion transfer in the TMB10 protein both K^+^ and Cl*^−^* ions experience repulsion at the first barrier region, in-dicating that spatial confinement is likely the main factor impeding their passage at this site. At the second site, situated at 5.15 nm along the chan-nel, the *K*^+^ ion encounters a potential energy barrier, while the *Cl^−^* ion is stabilized. Representative structures from the constraint simulations (Fig-ure S12) highlight the solvating water molecules and the first coordination sphere of protein sidechains. Both ions experience desolvation compared to the bulk in the region I. The total charge of sidechains in the first solvation shell is zero. Therefore, this barrier is dominated by the loss of solvating wa-ter molecules. In region II, on the other hand, both ions are fully solvated, but the net charge of the protein sidechains in the first solvation shell is +2, which leads to stabilization for the *Cl^−^* and destabilization of *K*^+^.

Even though experimentally there is no evidence that the TMB8 shows ion conductance, the free energy profiles for K^+^ and Cl*^−^* ions were determined for the constriction region only using thermodynamic integration as discussed for TMB10 and TMB12. The resulting PMF can be seen in Figure S13. There is a divergence at 3.2 nm Z position due to 3 positive Lys residues present at the opening to the pore, creating a significant initial barrier for K^+^ crossing into the channel. Both ions experience similar barriers at the gating region of 3.4-3.8 nm. Beyond 4 nm, the channel becomes more favorable to K^+^ than Cl*^−^*, due to the presence of internal negatively charged residues. The high barrier of 15 kcal/mol indicates that ion crossing is indeed unlikely for the TMB8 protein, which is consistent with the experimental results.

##### a

**b**

**I I**

**II**

**II**

**I**

**II**

5	5

4	4

3	3

**I**

**II**

∆G (kcal/mol)

pore radius (nm)

2	2

1	1

0	0

-1

-2

3.6	4	4.4	4.8	5.2	5.6

z (nm)

Figure S12: **Free energy of Cl***^−^* **and K**^+^ **transport through the TMB10**. **a**. Electrostatic potential along the pore channel in TMB10. Basic side chains are shown in blue and acidic in red. The channel sur-face was calculated using the HOLE program, see Methods for detail. **b**. Free energy of *Cl^−^* (red, circle) and *K*^+^ (blue, square) transport through the TMB10, calculated through thermodynamic integration. I,II show represen-tative structures of the ion *Cl^−^* (red) and *K*^+^ (blue) at the extrema of the free energy highlighting water molecules and protein side chains solvating the ion in the first coordination sphere.

16

12

∆G (kcal/mol)

8

4

0

3.2	3.6	4	4.4

z (nm)

Figure S13: **Potential of mean force (PMF) for TMB8 ion transport**. Free energy of *Cl^−^* (red) and *K*^+^ (blue) transport through the TMB8 protein, estimated from thermodynamic integration.

Table S2: **Simulation Details**. Number of lipid molecules, ions, and final dimensions of the simulation box.

| System | # POPC | # Ion | # Water | Final box dimensions  (nm^3^) |
| --- | --- | --- | --- | --- |
| TMB8 | 200 | 30 K^+^ / 27 Cl*^−^* | 10604 | 8.46*×*8.46*×*8.09 |
| TMB10 | 190 | 29 K^+^ / 29 Cl*^−^* | 11125 | 8.09*×*8.09*×*8.09 |
| TMB12 | 188 | 33 K^+^ / 30 Cl*^−^* | 11376 | 8.20*×*8.32*×*8.64 |
| TMB10 | 200 (DOPC) | 90 K^+^ / 90 Cl*^−^* | 32853 | 8.64*×*8.64*×*16.97 |
| TMB10 | 200 | 0 | 32853 | 8.64*×*8.64*×*19.97 |
| TMB10 + Glu | (DOPC,flow)  190 | 29 K^+^ / 29 Cl*^−^* | 11125 | 8.09*×*8.09*×*8.09 |
| Arabinose | 0 | 0 | 502 | 2.47*×*2.47*×*2.47 |
| Glucose | 0 | 0 | 878 | 2.97*×*2.97*×*2.97 |
| NAG | 0 | 0 | 875 | 2.97*×*2.97*×*2.97 |
| Sucrose | 0 | 0 | 2149 | 4.00*×*4.00*×*4.00 |
| Raffinose | 0 | 0 | 2141 | 4.00*×*4.00*×*4.00 |

# 5 Comparison between different types of ar-tificial membrane channels

Table S3: **Artificial membrane channels**. Advantages and disadvantages of different approaches for creating artificial membrane channels.

| **Channel platform** | **Advantages** | **Disadvantages** |
| --- | --- | --- |
| Artificial water channels  [3] | Structural simplicity,  high degree of synthetic control | High cost of achieving  functional diversity, lim-ited complexity |
| Carbon nanotube and boron nitride nanotube porins [4] | High permeability, ro-bust structure, poten-tial for precise control of channel diameter and wall properties | Low degree of synthetic control, limited mem-brane stability |
| *De novo* designed pro-tein pores [5] | Customizable structure, high specificity, func-tional versatility | Expression challenges, potential of misfolding |

# References

[1] Meni Wanunu, Tali Dadosh, Vishva Ray, Jingmin Jin, Larry McReynolds, and Marija Drndi´c. Rapid electronic detection of probe-specific micrornas using thin nanopore sensors. *Nat. Nanotechnol.*, 5(11):807–814, 2010.
[2] Ernest Awoonor-Williams and Christopher N. Rowley. Molecular simu-lation of nonfacilitated membrane permeation. *Biochim. Biophys. Acta, Biomembr.*, 1858(7, Part B):1672–1687, 2016.
[3] Yu Jie Lim, Kunli Goh, and Rong Wang. The coming of age of wa-ter channels for separation membranes: from biological to biomimetic to synthetic. *Chem. Soc. Rev.*, 51(11):4537–4582, 2022.
[4] Jie Shen, Gongping Liu, Yu Han, and Wanqin Jin. Artificial channels for confined mass transport at the sub-nanometre scale. *Nat. Rev. Mater.*, 6(4):294–312, 2021.
[5] Anastassia A. Vorobieva, Paul White, Binyong Liang, Jim E. Horne, Asim K. Bera, Cameron M. Chow, Stacey Gerben, Sinduja Marx, Alex Kang, Alyssa Q. Stiving, Sophie R. Harvey, Dagan C. Marx, G. Nasir Khan, Karen G. Fleming, Vicki H. Wysocki, David J. Brockwell, Lukas K. Tamm, Sheena E. Radford, and David Baker. De novo design of trans-membrane barrels. *Science*, 371(6531):eabc8182, 2021.
